# Multi-perspective dynamic consistency learning for semi-supervised medical image segmentation

**DOI:** 10.1038/s41598-025-03124-2

**Published:** 2025-05-25

**Authors:** Yongfa Zhu, Xue Wang, Taihui Liu, Yongkang Fu

**Affiliations:** College of Computer Science and Technology, Beihua University, Jilin, 132013 China

**Keywords:** Semi-supervised learning, Multi-perspective collaborative learning, Dynamic decoupling consistency, Model cognitive bias, Medical image segmentation, Computational models, Biomedical engineering, Bioinformatics, Computer science

## Abstract

Semi-supervised learning (SSL) is an effective method for medical image segmentation as it alleviates the dependence on clinical pixel-level annotations. Among the SSL methods, pseudo-labels and consistency regularization play a key role as the dominant paradigm. However, current consistency regularization methods based on shared encoder structures are prone to trap the model in cognitive bias, which impairs the segmentation performance. Furthermore, traditional fixed-threshold-based pseudo-label selection methods lack the utilization of low-confidence pixels, making the model’s initial segmentation capability insufficient, especially for confusing regions. To this end, we propose a multi-perspective dynamic consistency (MPDC) framework to mitigate model cognitive bias and to fully utilize the low-confidence pixels. Specially, we propose a novel multi-perspective collaborative learning strategy that encourages the sub-branch networks to learn discriminative features from multiple perspectives, thus avoiding the problem of model cognitive bias and enhancing boundary perception. In addition, we further employ a dynamic decoupling consistency scheme to fully utilize low-confidence pixels. By dynamically adjusting the threshold, more pseudo-labels are involved in the early stages of training. Extensive experiments on several challenging medical image segmentation datasets show that our method achieves state-of-the-art performance, especially on boundaries, with significant improvements.

## Introduction

Medical imaging plays a critical role in the clinical diagnostic process. Accurate segmentation of organs or lesions in medical images can assist physicians in rapidly diagnosing disease, assessing disease progression, and formulating treatment plans, including brain, abdomen, heart, and colon^1–5^. In recent years, deep learning has made significant achievements in medical image segmentation. However, the excellent capabilities of fully supervised data-driven methods need to rely on huge amount of labeled datasets^6–10^. Despite the large volume of image data available in the medical field, performing pixel-level data annotation requires considerable time and effort from experienced specialists to accomplish. Therefore, it is challenging for fully supervised learning methods to achieve superior segmentation performance with limited clinical high-quality labeled data^3,6,8,11^.

**Fig. 1:**
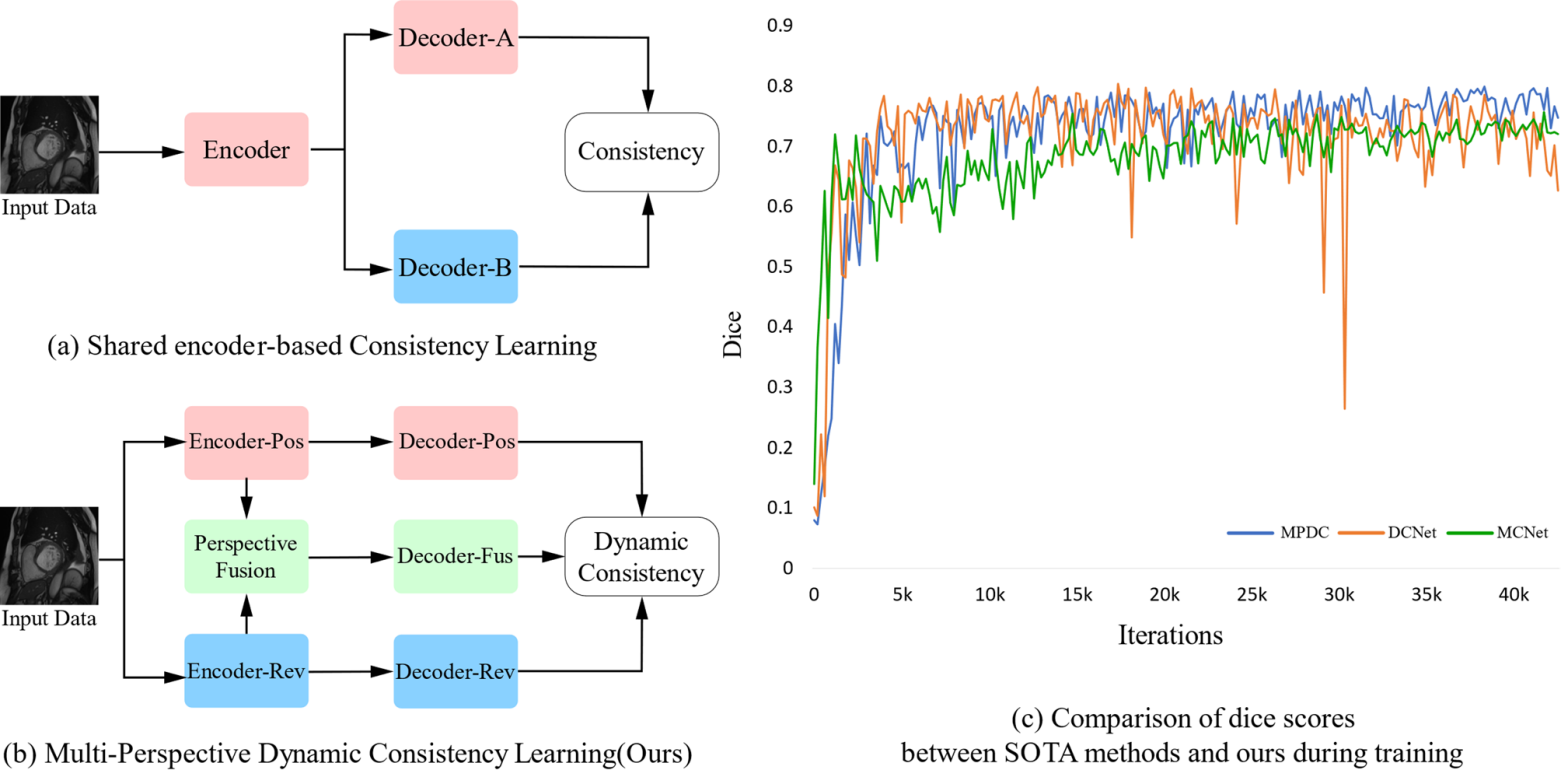
Comparison between SOTA methods and ours. (**a**) Traditional shared encoder-based consistency learning method. (**b**) Our proposed multi-perspective dynamic consistency learning method. (**c**) Comparison of dice scores with SOTA semi-supervised methods on the PROMISE12 dataset using 20% labeled data during training.

To overcome the limitations, semi-supervised learning methods have received wide attention, which utilize few pixel-level labeled data and large amounts of unlabeled data to train the network, effectively alleviating the problem of insufficient labeled data in medical image segmentation. Currently, a substantial amount of corresponding research has been carried out in semi-supervised medical image segmentation. Among them, pseudo-labeling^12–16^ and consistency regularization^17–21^ are two mainstream paradigms for semi-supervised learning. However, most of the existing consistency regularization-based methods train models based on a shared encoder and slightly distinct decoders, as shown in Fig. 1a. The above methods are prone to trap the model in cognitive bias, causing both branch networks to make incorrect predictions for the same region. Moreover, these incorrect predictions are difficult to rectify during the training process, which greatly affects the performance of the model. Analyzing the reason, it is mainly due to the deficiency of feature extraction ability between networks of similar structure for complex images. Facing the aforementioned issue, we come up with a new multi-perspective collaborative learning strategy that utilizes different branch networks to mine more informative features from distinct perspectives. As can be seen from Fig. 1b, the positive perspective branch focuses on learning features of the target region, whereas the reverse perspective branch focalizes on characteristics of the background region. Furthermore, to utilize these diverse features more comprehensively, a fusion perspective branch is implemented, which can fuse features from dual-perspective branches to present richer representational information, especially at the boundaries of the target objects. On the other hand, in consistency learning, traditional methods for acquiring high-confidence data use fixed thresholds, which may result in some important samples with low-confidence being ignored early in training, making the initial segmentation ability of the model insufficient, and leading to the accumulation of errors. Combining these limitations, we explore a dynamic decoupling consistency mechanism to fully utilize multi-perspective prediction data. To further demonstrate the advantages of our proposed multi-perspective dynamic consistency (MPDC) model, we compare it with state-of-the-art semi-supervised methods on the PROMISE12 dataset with 20% labeled data. As shown in Fig. 1c, we can see the Dice score of MCNet^22^ increases steadily during training, but it is not high. Although DCNet^23^ obtains a high Dice score, its growth curve is not stable and the Dice score even decreases during the training iterations. In contrast, the Dice score of our MPDC remains at a high level and grows more steadily.

In summary, to deal with the model cognitive bias due to insufficient feature extraction capability in complex medical images, and to fully utilize more pseudo-labels in the early training phase, we propose a multi-perspective dynamic consistency model for semi-supervised medical image segmentation, termed MPDC. First, we adopt multi-perspective branch framework for predicting segmentation regions, i.e., the positive perspective branch (for foreground prediction), the reverse perspective branch (for background prediction), and the fusion perspective branch (for boundary prediction), which can be trained collaboratively to calibrate the cognitive bias of the model and improve the ability of boundary perception. Second, we apply a dynamic decoupling consistency strategy to decouple the predicted data into consistent and inconsistent parts through dynamic threshold^23^. Third, to fully utilize the low-confidence data in the inconsistent part, we introduce the direction consistency loss to optimize the pixels near the decision boundary to be closer to the adjacent high-density region, thus making the decision boundaries clearer. In addition, we employ a pairwise cross-pseudo supervision strategy in the consistent part to fully utilize the above multi-perspective prediction information. The main contributions can be summarized as follows:

1. We propose a multi-perspective collaborative learning strategy to alleviate the problem of model cognitive bias caused by single-perspective consistency in semi-supervised medical image segmentation, and it enhances the capability of boundary perception through a Perspective Fusion Module.
2. We further introduce a dynamic decoupling consistency scheme to make full use of the multi-perspective prediction data. By dynamically adjusting the threshold, the predicted data is decoupled into a consistent part and an inconsistent part to effectively utilize the low-confidence pixels.
3. Extensive experiments are conducted on several challenging medical image segmentation datasets, including ACDC, PROMISE12, and two polyp segmentation datasets CVC-ClinicDB and Kvasir. The results prove that our method outperforms the SOTA semi-supervised segmentation methods, especially achieving significant improvements at the boundaries.

## Related work

### Deep semantic-based medical image segmentation

Semantic segmentation, categorized at the pixel level, is one of the key research directions in the field of medical image processing, which can accurately recognize anatomical structures and assist clinical diagnosis. U-Net^24^ is a pioneering work that proposed a fully convolutional encoder-decoder based architecture for performing pixel-level semantic segmentation in medical image, which successively inspired enormous works using similar architectures for more vision tasks^25–28^. Skip connections in U-Net can integrate low-level and high-level information from the encoding–decoding layer, improving the feature representation of the network. However, the semantic gap between low-level and high-level features may lead to introduce some redundant information in feature maps. Consequently, MultiResUNet^29^, MU-Net^30^, and FED-Net^31^ incorporated a convolutional layer-based attention mechanism between skip connections to alleviate the semantic gap problem and effectively enhanced segmentation performance. In addition, KiU-Net^32^ achieved excellent segmentation performance in small target area by cascading Ki-Net and U-Net, which offered fine edge feature maps and advanced shape feature maps respectively. While the above deep semantic learning methods have achieved exceptional performance in medical image segmentation, they rely heavily on pixel-level annotation supervision, which is often time-consuming and costly.

### Semi-supervised medical image segmentation

Semi-supervised learning (SSL) is a promising approach to reduce the model’s dependence on large-scale pixel-wise annotated data. It strives to train models using a small amount of labeled data and a large amount of unlabeled data. The core issue of the SSL method is how to fully utilize the vast unlabeled data. Recently, semi-supervised learning can be roughly categorized into two mainstream paradigms, i.e., pseudo-labeling^15,16,33–35^ and consistency regularization^20,21,36–38^. Pseudo-labeling methods involve using a model pre-trained on a small amount of labeled data to generate pseudo-labels for unlabeled data, which are then used to supervise model training. Consistency regularization-based methods employ the idea of encouraging the model to make the same prediction for different perturbations of the same image. Mean-Teacher model^39^ is a typical semi-supervised learning method that employs both pseudo-labeling and consistency regularization ideas. Unlike the teacher-student model, FixMatch^19^ trained the model by applying weak and strong augmentations to the input image and using the predictions from the weak augmentation to supervise the strong augmentation image. UniMatch^40^ further investigated the consistency between weak and strong augmentations in semi-supervised learning, expanding the space of data augmentation perturbations.

To further exploit pseudo-labels, CPS^14^ is proposed to facilitate mutual supervision of pseudo-labels generated simultaneously by two models, thereby achieving excellent performance. This approach has become a popular choice for pseudo-labeling and consistency regularization in many semi-supervised learning models. Alternatively, MCNet^22^ exploited two slightly distinct decoders and implemented cross-pseudo-supervision on the discrepancies between their outputs, encouraging mutual consistency to capture uncertain information. In addition, DMD^41^ enhanced the entropy of the prediction maps to increase the information content and performed consistency regularization on the entropy-augmented prediction maps.

Regarding pseudo-label selection, current models of pseudo-labeling and consistency regularization still rely on manually setting high threshold to select it. FlexMatch^42^ and FreeMatch^43^ pointed out that overly high threshold may discard many uncertain pseudo-labels, leading to imbalanced across-class learning and low pseudo-label utilization. Dynamic thresholding can utilize more pseudo-labels in the early training phase, but at the same time it inevitably introduces low-quality pseudo-labels. In SoftMatch^44^, a large number of high-quality pseudo-labels were maintained during training by assigning higher weights to high-confidence pseudo-labels and lower weights to low-confidence pseudo-labels.

To address the problem of model cognitive bias in semi-supervised learning, some approaches mitigated this problem from the perspective of consistency regularization constraints, such as co-training, by allowing two models to learn features from different perspectives. CCVC^17^ designed a co-training framework that forced two branches to learn informative features from different views. It introduced feature discrepancy loss to encourage the two networks to learn distinct features, and cross-pseudo supervision to ensure that the networks learn more information. Similar to co-training, LCVC^45^ considered local and global information to be complementary, and thus consistency constraints were applied to both local and global information, respectively. Unlike LCVC, MVANET^46^ performed multi-view complementary operations by fusing the global distant view with a local close-up view. This fusion helped to compensate for semantic deficiencies that may exist in a single view.

Compared with the above-mentioned methods, we propose a multi-perspective collaborative learning strategy to extract more comprehensive features from different perspectives, which effectively enhance the informative features of confusing regions and better understand the context of target objects at boundaries. In addition, a dynamic decoupling consistency mechanism is explored to take full advantage of multi-view prediction data.

## Method

**Fig. 2:**
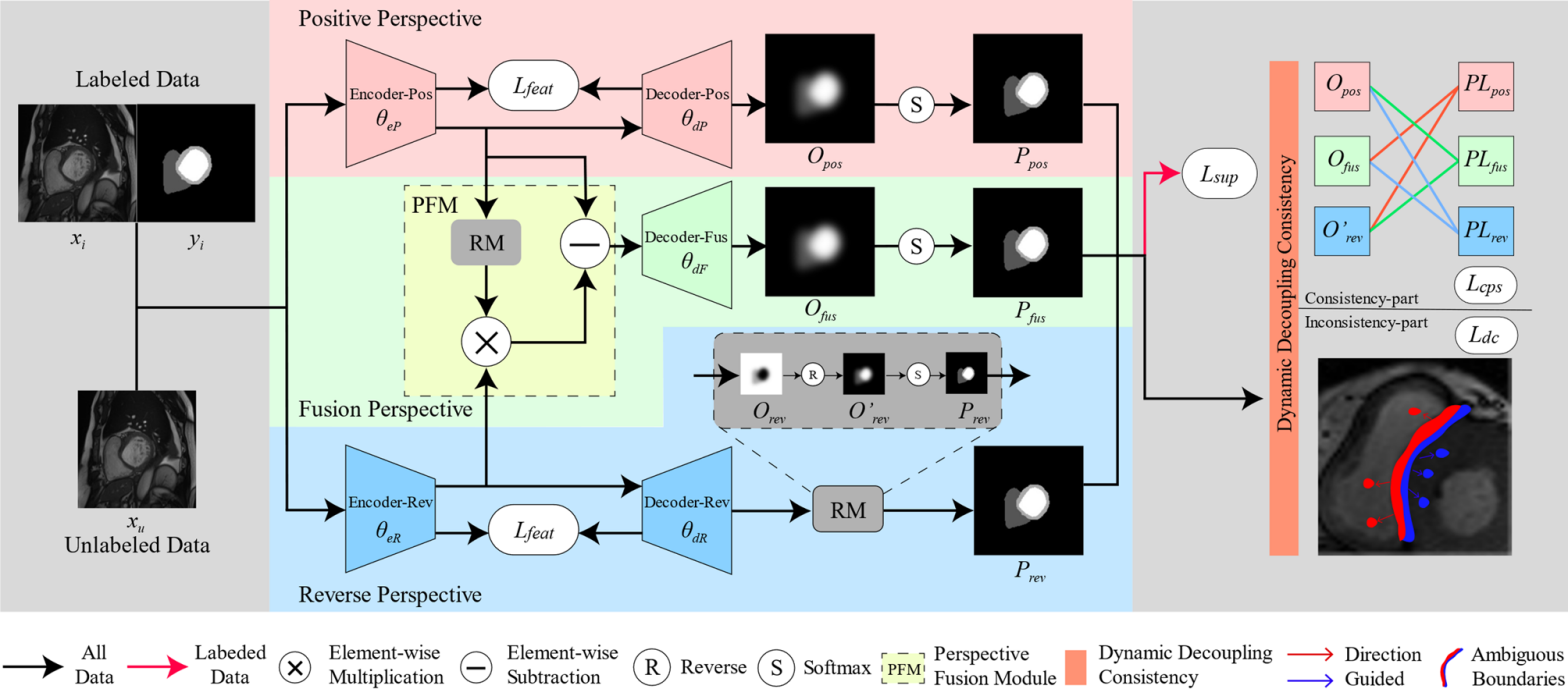
Framework of our proposed MPDC. MPDC contains three decoders $\theta_{dP}$, $\theta_{dR}$ and $\theta_{dF}$, where $\theta_{dP}$ uses bi-linear interpolation for up-sampling, $\theta_{dR}$ and $\theta_{dF}$ use transposed convolution for up-sampling. For the labeled data, we calculate the loss $L_{sup}$ between predictions and ground-truth. For the consistent part, we calculate cross pseudo supervision loss $L_{cps}$. For the inconsistent part, we calculate direction consistency loss $L_{dc}$. For feature maps between the encoders and decoders, we calculate the feature consistency loss $L_{feat}$.

Our designed multi-perspective dynamic consistency (MPDC) framework is shown in Fig. 2. The proposed architecture is composed of three parallel branches for multi-perspective collaborative learning. Specifically, we adopt two different perspective branches for feature extraction and segmentation prediction. The positive perspective branch is used to extract features of the target object areas and predict these areas, whereas the reverse perspective branch is utilized to extract features of the background areas and make predictions on them. To address the challenges in segmenting fuzzy boundaries, we implement a Perspective Fusion Module (PFM) to integrate the features extracted from the two perspective branches and improve the segmentation accuracy of the boundary region. Additionally, we employ the dynamic thresholds to categorize the predicted data into consistent and inconsistent parts. Regarding the consistent part, a cross-pseudo-supervision strategy is adopted among the predictions of unlabeled images from three branches for interactive optimization. Concerning the inconsistent part, the directional consistency is applied to fully harness the unlabeled data. Finally, in light of the significant difference between the positive and reverse perspective branches in the segmentation regions, we incorporate a feature consistency loss to strengthen the consistency constraint throughout the training of the encoders and decoders.

To precisely describe our method, we first define some mathematical terms. Let *D* = $D_{l}$ ∪ $D_{u}$ be the whole provided dataset. We denote an unlabeled image as $x_{u}$ ∈ $D_{u}$, a labeled image pair as ($x_{i}$, $y_{i}$) ∈ $D_{l}$ and $x \in x_{u} \cup x_{i}$, where $y_{i}$ is ground-truth. $\theta_{eP}$ and $\theta_{eR}$ represent the positive encoder and the reverse encoder, respectively. $\theta_{dP}$, $\theta_{dR}$ and $\theta_{dF}$ represent the positive decoder, the reverse decoder and the fusion perspective decoder, respectively. $\theta_{dP}$ employs bi-linear interpolation for up-sampling, while $\theta_{dR}$ and $\theta_{dF}$ employ original transpose convolution for up-sampling. The positive feature $f_{pos}$ and the reverse feature $f_{rev}$ can be obtained by $f_{pos} = \theta_{eP} \left( x \right)$ and $f_{rev} = \theta_{eR} \left( x \right)$, respectively.

### Multi-perspective collaborative learning

#### Positive perspective learning

In positive perspective branch, the positive feature $f_{pos}$ represents feature of target object regions in original image, which also represents the feature extraction perspective chosen by most deep neural networks. We denote the segmentation confidence map $P_{pos}$ as the positive prediction, which is the output $O_{pos}$ after softmax normalization, i.e., $P_{pos} = Softmax\left( {O_{pos} } \right)$. Specifically, the encoder $\theta_{eP}$ and the decoder $\theta_{dP}$ use U-net structure. Meanwhile, the output $O_{pos}$ is obtained by decoding the positive feature $f_{pos}$ through bilinear interpolation, which is defined as $O_{pos} = \theta_{dP} \left( {f_{pos} } \right)$. We apply Dice loss to fine-tune the positive perspective network, and the supervised loss can be defined as follows:

1 $$L_{{sup_{p} }} = Dice\left( {P_{pos} ,P_{gt} } \right)$$

where $L_{{sup_{p} }}$ represents the supervised loss from the positive perspective branch, $P_{gt}$ is ground-truth of the labeled image, i.e., $P_{gt} \in y_{i}$. It should be emphasized that only the labeled images $x_{i}$ are involved in the calculation of the supervised loss. The reverse perspective and the fusion perspective are also applicable.

#### Reverse perspective learning

Most of the existing models focus on learning features that are associated with the target region for image segmentation. However, unlike natural images, the contrast in medical images is low, the boundaries between different tissues or lesions are blurred, and the target regions and non-target regions (background region) are quite similar in terms of color and texture, which will yield a confusing result in these similar regions. The typical method of calculating loss based on target object regions may lead to insufficient attention to the background regions, resulting in the rich feature information in non-segmentation areas not being fully utilized during the encoding and decoding processes. In view of this, to leverage this overlooked feature space and extract more useful information, we develop the Reverse Perspective Learning (RPL) mechanism to teach the network to learn the features of non-target regions, thereby enhancing the influence of non-target region features on the model, and mitigating the cognitive bias of the model. Specifically, in our reverse perspective branch, the encoder $\theta_{eR}$ and the decoder $\theta_{dR}$ use the same U-net structure as the positive perspective. The difference is that transpose convolution is used in the decoder for up-sampling. Meanwhile, the network parameters of positive and reverse perspectives are not shared to prevent interfering with each other’s feature learning, which also avoids potential conflicts between them.

In addition, employing a reverse thinking approach, the output $O_{rev}$ is reversed by the Reverse Module (RM) before feeding it into the softmax normalization classifiers. Mathematically, the operation can be written as follows:

2 $$P_{rev} = Softmax\left( { - O_{rev} } \right)$$

where $O_{rev}$ is the output of the reverse perspective branch, which is defined as $O_{rev} = \theta_{dR} \left( {f_{rev} } \right)$. $P_{rev}$ denotes the segmentation confidence map after softmax normalization. The supervised loss of reverse perspective is formulated as below:

3 $$L_{{sup_{r} }} = Dice\left( {P_{rev} ,P_{gt} } \right)$$

From the above process, there are two possible inversion methods before calculating the reverse perspective branch loss: prediction map inversion and ground truth inversion. We adopt the former approach with the aim of maintaining the accuracy and integrity of the ground truth, and ensuring consistency with the predictions of the positive perspective branch during training.

#### Fusion perspective learning

**Fig. 3:**
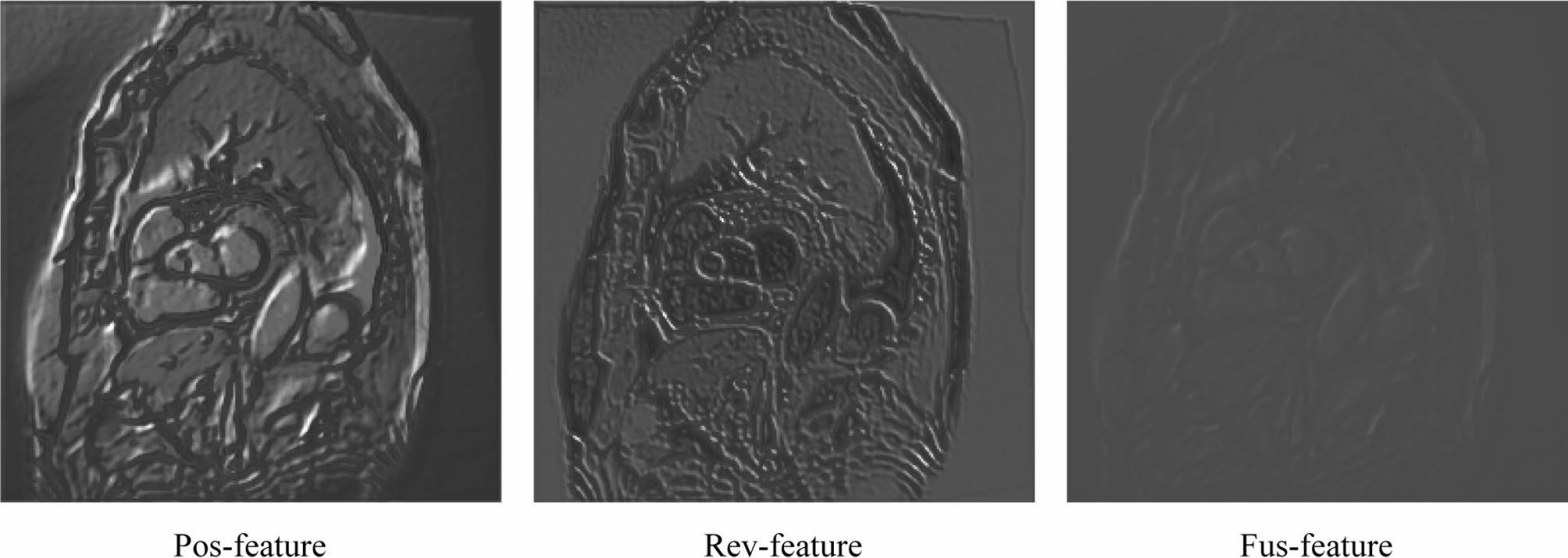
The feature maps generated from three perspectives. Among them, Pos-feature, Rev-feature and Fus-feature represent the feature maps generated by the positive perspective branch, the reverse perspective branch, and the fusion perspective branch, respectively.

The positive perspective branch and the reverse perspective branch focus on learning features of the target region and the background region, respectively, resulting in differences in the image features extracted by them. To utilize these diverse features more efficiently and comprehensively, we propose a Perspective Fusion Module (PFM). Specifically, by employing PFM, we can fuse image features from both positive and reverse perspectives to effectively enhance the informative features of confusing regions and better understand the context of target objects at boundaries. As shown in Fig. 3, the feature map yielded by PFM (Fus-feature) amplifies the response at the boundaries of confusing regions.

In fusion perspective branch, the PFM is introduced to fuse the image features from both positive and reverse perspectives. Specifically, through the Reverse Module (RM), the positive perspective feature map $f_{pos}$ is reversed and further normalized by softmax to obtain the feature map $f_{r - pos}$. Then, the feature map $f_{mul}$ is computed by pixel-wise multiplication of $f_{r - pos}$ and the softmax-normalized reverse perspective feature map $f_{rev}$. This purpose is to strengthen the consistent parts of the features from both positive and reverse perspectives, while weakening the inconsistent parts. Subsequently, the fused feature map $f_{fus}$ is obtained by pixel-wise subtraction of $f_{pos}$ and $f_{mul}$. After PFM, the role of the feature-consistent part in the fused feature map $f_{fus}$ will be weakened and the role of the feature-inconsistent part (e.g., confusing regions that often appear in the boundary region) will be amplified in the subsequent decoding process. Therefore, through the fusion perspective branch, the features of the object boundary can be effectively highlighted, which in turn improves the boundary segmentation accuracy of the target region. The Perspective Fusion Module is formulated as Eqs. (4)–(6):

4 $$f_{fus} = f_{pos} - f_{mul}$$

5 $$f_{mul} = f_{r - pos} \odot f_{rev}$$

6 $$f_{r - pos} = Softmax\left( { - f_{pos} } \right)$$

where $f_{fus}$ represents the feature map obtained from PFM, and $f_{r - pos}$ is the feature map obtained by reversing the positive perspective feature map $f_{pos}$ through RM and normalizing it.

Furthermore, through the decoder $\theta_{dF}$, the output $O_{fus}$ is derived from the fusion perspective branch, i.e., $O_{fus} = \theta_{dF} \left( {f_{fus} } \right)$. The segmentation confidence map $P_{fus}$ is acquired after softmax normalization, which is defined as $P_{fus} = Softmax\left( {O_{fus} } \right)$. The supervised loss of the fusion perspective is defined as follows:

7 $$L_{{sup_{f} }} = Dice\left( {P_{fus} ,P_{gt} } \right)$$

### Dynamic decoupling consistency

The consistency learning can reconcile three different perspectives so that their predictions converge. To optimize the prediction results, high-confidence maps are usually sampled as pseudo-masks for unlabeled data. However, traditional methods for acquiring high-confidence data use fixed thresholds, which may result in some important samples with low-confidence being overlooked early in training. In view of this, inspired by DCNet^23^, we employ a dynamic decoupling consistency strategy to fully utilize the advantages of the multi-perspective prediction data. Specifically, the dynamic threshold is used to compute pseudo-labels and decouple the predicted data into consistent part and inconsistent part. The dynamic threshold γ is defined as:

8 $$\gamma_{i} = \left\{ {\begin{array}{*{20}c} {\frac{1}{C},} & {i = 0} \\ {\min \left( {\gamma_{i}^{pos} ,\gamma_{i}^{rev} } \right),} & {i > 0} \\ \end{array} } \right.$$

9 $$\gamma_{i}^{pos/rev} = (1 - \lambda )\gamma_{{_{i - 1} }}^{pos/rev} + \lambda \frac{1}{B}\sum\nolimits_{b = 1}^{B} {{\text{max}}(P_{pos/rev} )}$$

where *C* and* B* represent the number of classes and the batch size, respectively. The weight coefficient $\lambda = \frac{i}{{i_{max} }}$ is used in the dynamic threshold calculation, where *i* is the number of iterations. $\gamma_{i}^{pos}$ and $\gamma_{i}^{rev}$ are the dynamic thresholds calculated from the segmentation confidence maps $P_{pos}$ and $P_{rev}$, respectively, as shown in Eq. (9). By comparing $\gamma_{i}^{pos}$ and $\gamma_{i}^{rev}$, we choose the smaller one as the final threshold to incorporate more unlabeled data in the training.

Further, the masks of consistent part and inconsistent part can be modeled as:

10 $$M = \left\{ {\begin{array}{*{20}l} {M_{con} ,} \hfill & {P_{pos} > \gamma \cap P_{rev} > \gamma } \hfill \\ {M_{incon} ,} \hfill & {otherwise} \hfill \\ \end{array} } \right.$$

where ${M}_{con}$ represents mask of consistent part, ${M}_{incon}$ represents mask of inconsistent part. γ is the dynamic threshold defined in Eq. (8), which allows more pixels to participate in the early stages of training and effectively improves data utilization.

#### Inconsistent part

In order to fully utilize the low-confidence data in the prediction results, according to the clustering assumption, the inconsistent part is further decoupled into confusing data (often found at decision boundaries) and guiding data (near high-density regions). Moreover, the direction consistency loss is introduced in this part to optimize the pixels near the decision boundary to be closer to the adjacent high-density region, thereby making the decision boundaries clearer. The high-confidence and low-confidence regions of the inconsistent part are described as follows:

11 $$P_{in - branch} = M_{incon} \odot P_{branch}$$

12 $$HM_{branch} = P_{in - branch} > P_{in - branch - diff}$$

13 $$hP_{branch} = HM_{branch} \odot P_{in - branch}$$

14 $$lP_{branch} = HM_{branch} \odot P_{in - branch - diff}$$

where the subscript *branch* represents positive (pos) or reverse (rev) perspective, and *branch-diff* indicates another perspective corresponding to the *branch*. $P_{in - branch}$ denotes inconsistent parts of confidence map in the positive or reverse perspective branch. $HM_{branch}$ represents high confidence mask of confidence map for inconsistent parts in positive or reverse perspective. We define the region that contrasts with the high-confidence region as the low-confidence region. Therefore, the inconsistent part is further decoupled into the high-confidence region $hP_{branch}$ and low-confidence region $lP_{branch}$. Ultimately, the direction consistency loss $L_{dc}$ can be formulated as:

15 $$L_{dc} = L2\left( {lP_{rev} ,detach\left( {hP_{pos} } \right)} \right) + L2\left( {lP_{pos} ,detach\left( {hP_{rev} } \right)} \right)$$

where *L*2 represents Euclidean distance loss.

#### Consistent part

Cross-pseudo supervision (CPS)^14^ is an effective semi-supervised consistency learning method designed to supervise networks through consistency of prediction results. For the consistent part decoupled from the predicted data, we employ a pairwise cross-pseudo supervision approach to fully utilize the prediction information from the three perspectives. The pairwise cross-pseudo supervision loss ${L}_{cps}$ is as follows:

16 $$L_{{cps}} = \left\{ {\begin{array}{*{20}l} {CE(hO_{{pos}} ,hPL_{{rev}} ) + CE(hO^{\prime} _{{rev}} ,hPL_{{pos}} ),} \hfill & {m < 0.95} \hfill \\ \begin{gathered} CE(O_{{pos}} ,PL_{{rev}} ) + CE(O^{\prime} _{{rev}} ,PL_{{pos}} ) + CE(O_{{pos}} ,PL_{{fus}} ) \hfill \\ \quad + CE(O_{{fus}} ,PL_{{pos}} ) + CE(O^{\prime} _{{rev}} ,PL_{{fus}} ) + CE(O_{{fus}} ,PL_{{rev}} ), \hfill \\ \end{gathered} \hfill & {m \ge 0.95} \hfill \\ \end{array} } \right.$$

where *CE*(.) is the cross-entropy loss function. $O^{\prime}_{rev}$ is the output of the reverse operation on $O_{rev}$ in RM. *m* stands for the mean confidence of $P_{pos}$ and $P_{rev}$, which we use as a judgment condition for using three-branch or two-branch cross-pseudo supervision. When* m* is less than 0.95, we only utilize the high-confidence part of $P_{pos}$ and $P_{rev}$ to generate corresponding pseudo-labels $hPL_{pos}$ and $hPL_{rev}$, considering that the pseudo-label generated by the average confidence map is unreliable. On the contrary, when *m* is greater than 0.95, which indicates that the average confidence map is reliable, and we utilize the whole part of $P_{pos}$, $P_{rev}$ and $P_{fus}$ to generate corresponding pseudo-labels $PL_{pos}$, $PL_{rev}$, and $PL_{fus}$.

### Loss function

#### Feature consistency loss

The feature maps obtained from both positive and reverse perspectives contain rich complementary information, and to fully utilize these features and ensure that they remain consistent throughout the encoding–decoding process, we introduce the feature consistency loss^23^. In addition, to reduce the training pressure on model parameters, channel compression is employed to the feature maps. The channel compression is defined as follows:

17 $$\overline{f}_{m} = \frac{1}{C}\sum\limits_{i}^{C} {\left| {f_{mi} } \right|^{p} }$$

where $f_{m}$ represents the feature map of the *m*-th layer, and $f_{mi}$ represents the *i*-th slice of $f_{m}$ along the channel dimension. $\overline{f}_{m}$ denotes the mapping result. *C* is the number of channels. *p* stands for the hyperparameter, which is set to 2. Its purpose is to increase the entropy of each channel in each layer of the feature map, thus ensuring that more feature information is carried during the feature fusion process. The feature consistency loss can be expressed as below:

18 $$L_{feat} = \sum\limits_{m = 1}^{n} {\sum\limits_{i = 1}^{N} {\left| {\left| {\overline{f}_{mi}^{e} - \overline{f}_{mi}^{d} } \right|} \right|_{2}^{2} } }$$

where *N* represents the number of pixels in $\overline{f}_{m}$, and *n* represents the number of layers in the network. $\overline{f}_{mi}^{e}$ and $\overline{f}_{mi}^{d}$ denote the *i*-th pixel of the *m*-th feature map of the encoder and decoder, respectively. In this paper, the loss also applies to the reverse perspective branch.

#### Total loss

The final loss function of our network consists of two parts: the supervised loss and the unsupervised loss. The supervised loss includes common segmentation loss derived from three distinct perspectives. It can be formalized as follows:

19 $$L_{sup} = L_{{sup_{p} }} + L_{{sup_{r} }} + L_{{sup_{f} }}$$

The unsupervised loss is a combination of the pairwise cross-pseudo supervision loss in consistent part and the direction consistency loss in inconsistent parts and the feature consistency loss. Ultimately, the total loss $L$ is defined as follows:

20 $$L = L_{sup} + L_{cps} + L_{dc} + L_{feat}$$

## Experiment and results

### Datasets and evaluation metrics

Our method is evaluated on ACDC^47^, PROMERE12^48^ and polyp datasets of CVC-ClinicDB and Kvasir^49^. In the ACDC dataset, we randomly select 140 scans from 70 subjects for the training set, 20 scans from 10 subjects for the validation set, and 40 scans from 20 subjects for the test set, ensuring that each set contains data from different subjects. In the PROMISE12 dataset, we randomly divide the data into 35, 5, and 10 cases for training, validation, and testing. For the polyp datasets of CVC-ClinicDB and Kvasir, we divide them into the training set, validation set and test set according to the ratio of 7:1:2, consisting of 1015 images for training, 145 images for validation, and 290 images for testing. Besides, according to the prior work on datasets^50^, the above datasets are segmented in 2D. Each slice is resized to 256 × 256, and the pixel intensities are normalized to the range [0, 1]. We use four standardized evaluation metrics, i.e., the Dice Similarity Coefficient (Dice), the Jaccard Similarity Coefficient (Jaccard), the 95th percentile Hausdorff Distance (95HD) and the Average Surface Distance (ASD), where Dice and Jaccard are used for evaluating overall prediction accuracy, and 95HD and ASD are used for evaluating the accuracy of boundary prediction.

### Implementation details

To ensure fair comparisons, we use the same experimental setup for comparison and ablation experiments, which are conducted on PyTorch using an NVIDIA GeForce RTX 2080 Ti GPU. We utilize U-net^24^ as our baseline network. And SGD is used as an optimizer with a weight decay of 0.0005 and a momentum of 0.9. In addition, the learning rate is 0.01 and the batch size is set to 24, where 12 images are labeled. During training, 50,000 iterations are performed for all methods. Moreover, we employ a data augmentation strategy including random flipping and random rotation to alleviate the overfitting problem. Our code is available at https://github.com/SoulRuolan/MPDC-net.

### Comparison with state-of-the-art methods

To demonstrate the advancements of our method, we compare it with state-of-the-art semi-supervised methods, including URPC^18^, MCNet^22^, SSNet^50^, DCNet^23^. The metrics of Dice, Jaccard, 95% Hausdorff Distance (95HD), and Average Surface Distance (ASD) are employed to evaluate the results of three segmentation tasks.

#### Results on ACDC

Derived from the MICCAI 2017 challenge, ACDC is a cardiac dynamic magnetic resonance imaging dataset designed to segment the left ventricle (LV), right ventricle (RV), and myocardium (Myo) in MRI. Table 1 shows the performance of three-class segmentation including Myo, LV and RV on the ACDC dataset. First, our method is compared with the U-Net baseline, which is used as a lower standard for semi-supervised methods. Compared to a supervised-only baseline, our method improves 10.87%, 14.92%, 9.52 and 2.81 for Dice, Jaccard, 95HD and ASD, respectively. This suggests that our approach can effectively utilize unlabeled data to improve the ability of the network to learn the overall data distribution. Furthermore, compared to other state-of-the-art semi-supervised methods, our method achieves optimal performance on all metrics using only 10% of the labeled data, improving by 0.82%, 1.33%, 0.07, and 0.77 points, respectively. It also demonstrates that our method is effective in reducing the labor consumption associated with the annotation of training data.

**Table 1:** Comparison with state-of-the-art methods on the ACDC dataset.

Method	#Scans		Metrics			
	Labeled	Unlabeled	Dice (%)↑	Jaccard (%)↑	95HD (voxel)↓	ASD (voxel)↓
SupOnly	7 (10%)	63 (90%)	79.37	67.78	10.73	3.12
URPC^18^			83.10	72.41	4.84	1.53
MCNet^22^			86.44	77.04	5.50	1.84
SSNet^50^			86.78	77.67	6.07	1.40
DCNet^23^			89.42	81.37	1.28	0.38
MPDC (Ours)			90.24	82.70	1.21	0.31

**Fig. 4:**
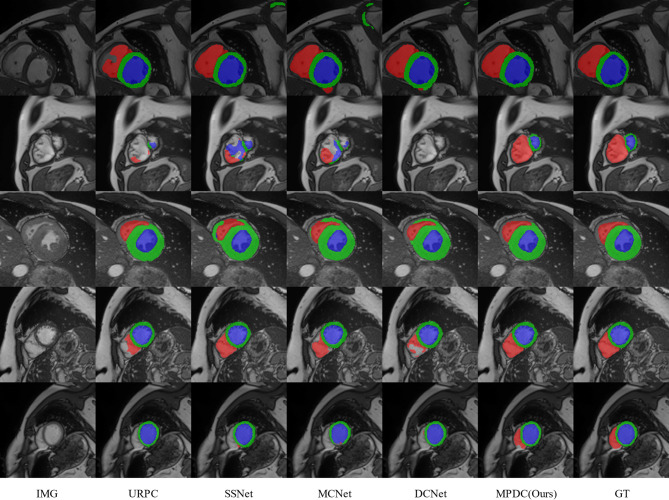
Visual comparison of different methods on the ACDC dataset.

We also provide a visual comparison of the above semi-supervised methods using 7 labeled examples on the ACDC dataset. As shown in Fig. 4, our method can generate sufficiently accurate segmentation results for different slices of left ventricle (LV), right ventricle (RV), and myocardium (Myo). Compared to other semi-supervised methods, our approach exhibits more accurate segmentation capability in terms of boundary segmentation, which also proves the effectiveness of our multi-perspective collaborative learning strategy. Other semi-supervised segmentation methods produce inaccurate results to varying degrees when segmenting the above three regions. And our method can effectively ameliorate this problem, thus obtaining more anatomically realistic results.

#### Results on PROMISE12

PROMISE12 is an open source prostate magnetic resonance imaging segmentation dataset. Table 2 shows the comparison results on the PROMISE12 dataset, where 20% of the labeled data from the training samples are used. From Table 2, we can see that our method still achieves the optimal segmentation performance compared to other state-of-the-art semi-supervised methods, which indicates the robustness of our method. Compared with the supervised-only U-Net baseline, our method significantly improves the Dice, Jaccard, 95HD, and ASD metrics by 16.74%, 16.03%, 9.49 and 2.06, respectively. Moreover, compared to other semi-supervised methods, our approach improves on all metrics by 0.34%, 1.01%, 3.59 and 1.03, respectively. In particular, the 95HD and ASD metrics are significantly improved, which further validates the segmentation capability of our method in the edge region.

**Table 2:** Comparison with state-of-the-art methods on the PROMISE12 dataset.

Method	#Scans		Metrics			
	Labeled	Unlabeled	Dice (%)↑	Jaccard (%)↑	95HD (voxel)↓	ASD (voxel)↓
SupOnly	7 (20%)	28 (80%)	62.28	50.42	16.55	3.56
URPC^18^			67.04	54.01	11.54	2.11
MCNet^22^			71.77	59.07	10.76	2.85
SSNet^50^			71.56	59.35	14.38	3.03
DCNet^23^			78.68	65.44	10.65	2.53
MPDC (Ours)			79.02	66.45	7.06	1.50

**Fig. 5:**
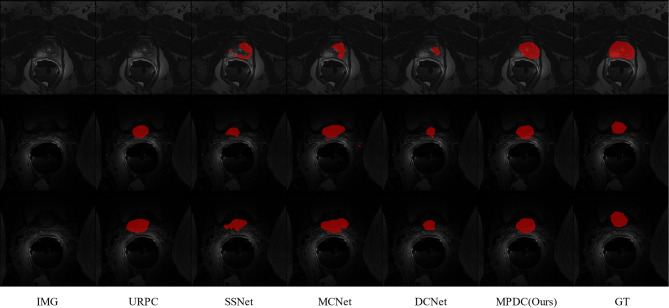
Visual comparison of different methods on the PROMISE12 dataset.

To visualize the effect of prostate segmentation in the PROMISE12 dataset, we also perform a visual comparison of the semi-supervised methods listed in Table 2. As shown in Fig. 5, the edges of the prostate in the PROMISE12 dataset are blurred and have low contrast with the background region, making it more difficult to segment. Other semi-supervised segmentation methods are ineffective in segmenting the prostate, with some degree of under- or over-segmentation. However, our method compensates for this deficiency to some extent by introducing a Perspective Fusion Module (PFM), which can greatly improve the segmentation accuracy. Also, our proposed multi-perspective collaborative learning mechanism can improve both the interior and edges of the segmented anatomy.

#### Results on CVC-ClinicDB and Kvasir

The experiments demonstrate that our method also produces excellent results on two polyp datasets of CVC-ClinicDB and Kvasir, which further illustrates the robustness of our method. Table 3 shows the comparison results of the different methods on the CVC-ClinicDB and Kvasir. As can be seen from the table, our method still outperforms the others on the four evaluation indicators. Compared to the supervised-only U-Net baseline, our method improves 9.9%, 12.64%, 33.06 and 12.86 for Dice, Jaccard, 95HD and ASD, respectively. In addition, compared with other state-of-the-art semi-supervised methods, our method also improves 1.52%, 2.13%, 10.14 and 4.46 in Dice, Jaccard, 95HD and ASD, respectively. Especially, our method still exhibits excellent performance in terms of boundary segmentation capability. This proves that our proposed semi-supervised method achieves good performance in medical image segmentation tasks of different modalities.

**Table 3:** Comparison with state-of-the-art methods on the CVC-ClinicDB and Kvasir.

Method	#Scans		Metrics			
	Labeled	Unlabeled	Dice (%)↑	Jaccard (%)↑	95HD (voxel)↓	ASD (voxel)↓
SupOnly	102 (10%)	913 (90%)	69.70	59.28	63.60	15.77
URPC^18^			69.55	59.75	61.23	14.88
MCNet^22^			70.48	61.05	61.47	13.38
SSNet^50^			73.27	62.97	63.07	16.42
DCNet^23^			78.08	69.79	40.68	7.37
MPDC(Ours)			79.60	71.92	30.54	2.91

**Fig. 6:**
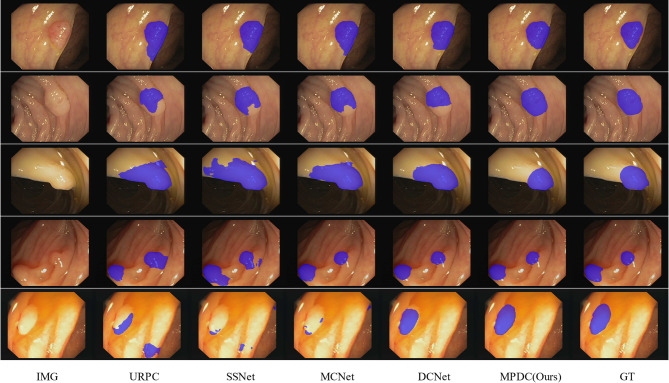
Visual comparison of different methods on the polyp dataset of CVC-ClinicDB and Kvasir.

We also provide a visual comparison of the semi-supervised methods listed in Table 3 on the polyp dataset of CVC-ClinicDB and Kvasir. As shown in Fig. 6, compared with other semi-supervised methods, our method can obtain more accurate polyp regions in various complex segmentation scenarios such as uneven brightness, blurred boundaries, etc., on the CVC-ClinicDB and Kvasir datasets where the labeling rate is only 10%. Thus, the experimental results further show that our method can achieve better segmentation performance with a small number of annotations.

### Ablation study

In this section, we further analyze the effectiveness of each strategy in our approach, including multi-perspective collaborative learning and dynamic decoupling consistency. Here, we perform ablation experiments on the ACDC and PROMISE12 datasets.

#### Effectiveness of multi-perspective collaborative learning

To verify the contribution of multi-perspective collaborative learning strategy, we conduct experiments on the ACDC and PROMISE12 datasets with 10% and 20% labeled data respectively. The multi-perspective collaborative learning strategy consists of three branches, i.e., positive perspective branch, reverse perspective branch, and fusion perspective branch. The positive perspective branch focuses on learning features of the target region, whereas the reverse perspective branch focuses on learning features of the background region. They can enrich the learned image features from different viewpoints. In addition, to more effectively and comprehensively utilize these diverse features to further exploit the capabilities of network learning, a fusion perspective branch is introduced. The experimental results are shown in Table 4.

**Table 4:** Ablation studies of multi-perspective branch on ACDC dataset with 10% labeled data and PROMISE12 dataset with 20% labeled data.

Branch1	Branch2	Branch3	ACDC				PROMISE 12			
			Dice (%)↑	Jaccard (%)↑	95HD (voxel)↓	ASD (voxel)↓	Dice (%)↑	Jaccard (%)↑	95HD (voxel)↓	ASD (voxel)↓
Pos	Rev	–	89.57	81.70	1.42	0.47	76.86	64.24	7.59	1.52
Pos	Pos	Fus	89.67	81.84	1.94	0.47	77.59	64.84	**6.64**	1.53
Pos	Rev	Fus	**90.24**	**82.69**	**1.21**	**0.31**	**79.02**	**66.45**	7.06	**1.50**

The positive perspective branch is denoted as Pos, the reverse perspective branch is denoted as Rev, and the fusion perspective branch is denoted as Fus.

Significant values are in bold.

It can be seen from Table 4 that using three perspective branches (Pos + Rev + Fus) in the ACDC segmentation task improves the metrics of Dice, Jaccard, 95HD and ASD by 0.67%, 0.99%, 0.21, and 0.16, respectively. In addition, on the PROMISE12 dataset, Dice, Jaccard and ASD metrics also improved by 2.16%, 2.21%, and 0.02 respectively. This demonstrates the effectiveness of multi-perspective collaborative learning in different semi-supervised segmentation tasks. To further verify the validity of the reverse perspective branch, we also conduct comparative experiments between Pos + Pos + Fus and Pos + Rev + Fus. Compared to Pos + Pos + Fus, our method achieves better and more stable results on most of the metrics for both datasets, which shows a significant improvement in reverse perspective learning.

In the reverse perspective branch, we also compare two inversion methods for feature extraction, i.e., prediction map inversion and ground truth inversion. As shown in Table 5, employing the prediction map inversion method in reverse perspective learning achieves consistent performance gains in all metrics, with Dice, Jaccard, 95HD and ASD improving by 0.75%, 1.16%, 2.85, and 0.49, respectively. This demonstrates the importance of maintaining the completeness of the ground truth. Moreover, taking the prediction map inversion approach is more conducive to the network learning diverse features.

**Table 5:** Comparison of two inversion methods in the reverse perspective branch on ACDC dataset with 10% labeled data, i.e., prediction map inversion (denoted as PM_Inver) and ground truth inversion (denoted as GT_Inver).

Method	Dice (%)↑	Jaccard (%)↑	95HD (voxel)↓	ASD (voxel)↓
GT_Inver	89.49	81.53	4.06	0.80
PM_Inver	90.24	82.69	1.21	0.31

In addition, we also conduct a comparative experiment of shared encoder. As shown in Table 6, sharing the encoder between the positive perspective and the reverse perspective can reduce the number of network parameters to a certain extent, but the segmentation performance of the model decreases. As can be seen from Table 6, on the three segmentation datasets, the Dice, Jaccard, 95HD, and ASD metrics in our parameter non-sharing method have achieved significant improvements. The main reason is that parameter non-sharing method can effectively prevent the interference of feature learning between each other, which also avoids potential conflicts between them.

**Table 6:** Ablation studies of shared encoder for MPDC.

Encoder	Dataset	Metrics					
		Dice (%)↑	Jaccard (%)↑	95HD (voxel)↓	ASD (voxel)↓	FLOPs (GMac)	Parameters (M)
Shared	ACDC	88.76	80.45	1.41	0.40	7.80	3.34
Individual		**90.24**	**82.70**	**1.21**	**0.31**	8.89	4.52
Shared	PROMISE12	76.07	62.41	29.25	5.85	–	–
Individual		**79.02**	**66.45**	**7.06**	**1.50**	**–**	**–**
Shared	Polyp	77.94	69.31	39.50	7.97	–	–
Individual		**79.60**	**71.92**	**30.54**	**2.91**	**–**	**–**

Where ‘Shared’ denotes that the positive perspective and reverse perspective share the same encoder, ‘Individual’ indicates that the positive perspective and reverse perspective use a separate encoder.

Significant values are in bold.

#### Effectiveness of dynamic decoupling consistency

To further explore the effectiveness of dynamic decoupling consistency, we conduct comparative experiments between dynamic thresholding and traditional fixed thresholding. As shown in Table 7, our dynamic thresholding method yields superior performance compared to fixed thresholding. When the fixed threshold is set to 0.5, the model performs worst, primarily because more uncertain pixels are incorporated in the prediction results at this threshold. Although the thresholds of 0.2 and 0.9 achieve better performance, some important samples with low-confidence are still omitted early in training. In contrast, the prediction data from multiple perspectives can be effectively utilized by employing a dynamic thresholding approach.

**Table 7:** Ablation studies of threshold selection methods for the decoupling consistency section on ACDC dataset with 10% labeled data.

Threshold	Dice (%)↑	Jaccard (%)↑	95HD (voxel)↓	ASD (voxel)↓
0.25	89.33	81.23	2.85	0.71
0.50	87.79	78.91	5.95	1.72
0.90	89.81	81.02	1.38	0.32
Dynamic(ours)	90.24	82.69	1.21	0.31

We also conduct experiments in Table 8 to validate the effect of pairwise cross-pseudo supervision among three different perspectives on the ACDC dataset with 10% labeled data. As can be seen from Table 8, by introducing the fusion perspective for cross-pseudo supervision, the value of the 95HD is improved by 0.47, which indicates that the fusion perspective has an advantage in boundary segmentation capability. However, the overall segmentation performance is not satisfactory, with Dice and Jaccard decreasing by 0.92% and 1.26%, respectively. Indeed, the overall segmentation ability of cross-pseudo supervision between the positive and reverse perspectives complements the boundary segmentation ability of the fusion perspective. Therefore, we employ pairwise cross-pseudo supervision among three perspectives. It can be seen that our method performs optimally on all metrics.

**Table 8:** Ablation studies of multi-perspective cross-pseudo supervision (CPS) in consistent part on ACDC dataset with 10% labeled data.

P–R	P–F	R–F	Dice (%)↑	Jaccard (%)↑	95HD (voxel)↓	ASD (voxel)↓
√			90.06	82.37	1.78	0.40
	√	√	89.14	81.11	1.31	0.50
√	√	√	90.24	82.69	1.21	0.31

Where P–R represents CPS between the positive and the reverse perspectives, P–F represents CPS between the positive and the fusion perspectives, and R–F indicates CPS between the reverse and the fusion perspectives.

## Conclusion

In this work, we design a novel semi-supervised method based on multi-perspective dynamic consistency for medical image segmentation. The multi-perspective collaborative learning strategy is employed to enrich the informative features and prevent model cognitive bias due to the insufficient feature extraction capability from a single viewpoint. Meanwhile, a dynamic decoupling consistency scheme is introduced to efficiently utilize low-confidence pixels by dynamically adjusting the thresholds so that more pseudo-labels are involved in the training process. Additionally, we adopt an integrated loss that facilitates collaborative learning of multi-branch networks from different perspectives. Experimental verifications conducted on ACDC, PROMISE12 and two polyp datasets of CVC-ClinicDB and Kvasir show that our method outperforms other semi-supervised segmentation approaches. Furthermore, we perform a series of ablation studies to validate the effectiveness of our proposed components in the model. Although our method achieves new SOTA performance, it tends to underperform in some challenging segmentation tasks, especially when the number of labeled samples is small. Our multi-perspective learning strategy learns a broader range of features, which implies the ability to capture more details. However, due to the inherent characteristics of complex images, an increase in the number of features may lead to overlearning of the model, thereby degrading its performance. In the future, we will further investigate the effective feature selection of the model to better take advantage of multi-perspective learning, and improve the robustness and generalization of the model.

## Data Availability

The datasets used in the study are publicly available at the following links: ACDC—https://www.creatis.insa-lyon.fr/Challenge/acdc/. PROMISE12—https://promise12.grand-challenge.org/Home/. polyp datasets of CVC-ClinicDB and Kvasir—https://github.com/DengPingFan/PraNet.
